# Pentraxin-3 modulates hepatocyte ferroptosis and the innate immune response in LPS-induced liver injury

**DOI:** 10.1186/s43556-024-00227-6

**Published:** 2024-12-12

**Authors:** Huitong Wang, Zhaojie Su, Yunyun Qian, Baojie Shi, Hao Li, Wenbin An, Yi Xiao, Cheng Qiu, Zhixiang Guo, Jianfa Zhong, Xia Wu, Jiajia Chen, Ying Wang, Wei Zeng, Linghui Zhan, Jie Wang

**Affiliations:** 1 Department of Organ Transplantation, School of Medicine, Organ Transplantation Clinical Medical Center of Xiamen University, Xiang’an Hospital of Xiamen University, Xiamen University, Xiamen, Fujian 361102 China; 2 Xiamen Human Organ Transplantation Quality Control Center, Xiamen Key Laboratory of Regeneration Medicine, Fujian Provincial Key Laboratory of Organ and Tissue Regeneration, School of Medicine, Organ Transplantation Institute of Xiamen University, Xiamen University, Xiamen, Fujian 361102 China; 3 Department of Gastroenterology, School of Medicine, Xiang’an Hospital of Xiamen University, Xiamen University, Xiamen, Fujian 361102 China; 4 Department of Critical Care Medicine, Zhongshan Hospital Affiliated to Xiamen University, Xiamen, Fujian 361004 China

**Keywords:** PTX3, LPS, Liver, Crosstalk, Macrophage polarization

## Abstract

The liver plays a crucial role in the immune response during endotoxemia and is one of the critical targets for sepsis-related injuries. As a secretory factor involved in inflammation, pentraxin-3 (PTX3) has been demonstrated to regulate hepatic homeostasis; however, the relationship between PTX3 and cell crosstalk between immune cells and hepatocytes in the liver remains incompletely understood. In this study, we revealed that, compared with WT mice, *Ptx3*^−/−^ mice with lipopolysaccharide (LPS)-induced endotoxemia exhibited alleviated liver damage, with reduced serum alanine transaminase and aspartate transaminase levels and an improved survival rate. Mechanistically, RNA-Seq and western blot results revealed that *Ptx3* knockdown in hepatocytes increased the expression of *Tfrc* and *Ccl20*; consequently, *Ptx3* deficiency regulated LPS-induced hepatocyte ferroptosis via increased mitochondrial reactive oxygen species and Fe^2+^ and recruited more macrophages by CCL20/CCR6 axis to be involved in inflammation and the clearance of harmful substances. Moreover, western blot and immunofluorescence staining confirmed that the NF-κB signaling pathway was upregulated upon LPS treatment in *Ptx3*-knockdown macrophages, promoting phagocytosis and polarization toward M1 macrophages. Collectively, our findings show that the absence of *Ptx3* can ameliorate sepsis-induced liver injury by regulating hepatocyte ferroptosis and promote the recruitment and polarization of M1 macrophages. These findings offer a key basis for the development of effective treatments for acute infections.

**Supplementary Information:**

The online version contains supplementary material available at 10.1186/s43556-024-00227-6.

## Introduction

Sepsis is one of the most lethal infectious diseases, characterized by excessive systemic inflammation and multiorgan failure, responsible for 20% of annual disease deaths [1, 2]. The liver constitutes a frontline defense and suffers the earliest and most severe injury when sepsis occurs. Thus, liver dysfunction represents an early injury event in sepsis and has been identified as a strong predictor of poor prognosis in the clinic [3]. Therefore, elucidating the underlying mechanisms of sepsis-induced liver injury is important to limit infection and improve survival in patients with sepsis.

As the liver is the primary organ involved in the storage and metabolism of iron in the body, liver damage is intricately linked to iron overload. Uncontrolled levels of free iron have toxic effects on the liver, leading to ferroptosis of hepatocytes and exacerbating liver failure after treatment with lipopolysaccharide (LPS), and an increasing number of studies have reported a link between ferroptosis and sepsis [4, 5]. Ferroptosis represents a novel form of programmed cell death that relies on excessive iron and reactive oxygen species (ROS) stemming from lipid peroxidation. The upregulation of the expression of transferrin receptor (TFRC), a key iron transporter protein in the cell membrane of hepatocytes, enhances iron uptake and promotes the accumulation of unstable iron pools, thereby triggering ferroptosis [6]. Consequently, TFRC has emerged as a pivotal regulator of cellular ferroptosis. Inhibiting TFRC dramatically augments infection by mitigating lipid peroxidation, indicating potential resistance to infection with increased ferroptosis [7].

Many studies have highlighted the critical role of the ferroptosis of hepatocytes in orchestrating a cascade of immune responses during endotoxemia [8–10].

Ferroptotic cells have also been shown to induce the recruitment of macrophages through the regulation of inflammation-related genes [11, 12]. As pivotal immune components within the liver, macrophages form the first line of defense for recognizing and eliminating microorganisms, thereby safeguarding the body against infections [13, 14]. Upon intravenous bacterial infection, macrophages swiftly capture more than 60% of the bacteria in the liver within 10 min, with this proportion increasing to more than 80% within 6 h [15]. Macrophages polarize toward the proinflammatory M1 phenotype, characterized by the surface overexpression of CD86 and CD68 and the release of proinflammatory factors such as tumor necrosis factor-α (TNF-α) and interleukin-1 beta (IL-1β) to bolster the host’s defense against bacteria [16]. However, the intricate communication between ferroptotic hepatocytes and macrophages during endotoxemia remains poorly understood.

Pentraxin-3 (PTX3) was firstly identified as a member of the long pentraxin subfamily and is characterized by a long, unrelated N-terminal domain and a C-terminal pentraxin domain. It is secreted by monocytes and macrophages [17]. Despite emerging evidence indicating that PTX3 is a robust prognostic predictor of sepsis [18, 19], its role in resistance against bacterial infection remains controversial. PTX3 has been shown to modulate LPS-induced inflammation in human primary liver macrophages and peripheral monocytes by favoring the M2 phenotype [20]. Additionally, exogenous PTX3 has been found to increase the production of the anti-inflammatory cytokine IL-10 in human peripheral blood [21]. PTX3 was also proven to have a proinflammatory effect, as evidenced by the significant suppression of the expression of IL-6, IL-8, and IL-1β upon *Ptx3* silencing [22]. Compared with wild-type mice, *Ptx3*-transgenic mice are more resistant to LPS toxicity and to cecal ligation and puncture [23]. Therefore, investigating the function of PTX3 in the genesis and development of LPS-induced liver injury is necessary.

In this study, we observed an increased level of PTX3 in the serum during sepsis. Mechanistically, our result showed that *Ptx3* inhibition ameliorated liver damage through the induction of hepatocyte ferroptosis and the recruitment of M1-type macrophages by CCL20/CCR6 axis and the polarization of M0-type to M1-type by the activation of the NF-κB pathway. The findings contribute significantly to our understanding of the role of PTX3 in liver injury and innate immunity, potentially opening new avenues for sepsis treatment. Thus, PTX3 could be a potential prognostic indicator and therapeutic target in sepsis.

## Results

### PTX3 deficiency alleviates LPS-induced liver injury and inflammation

**Fig. 1:**
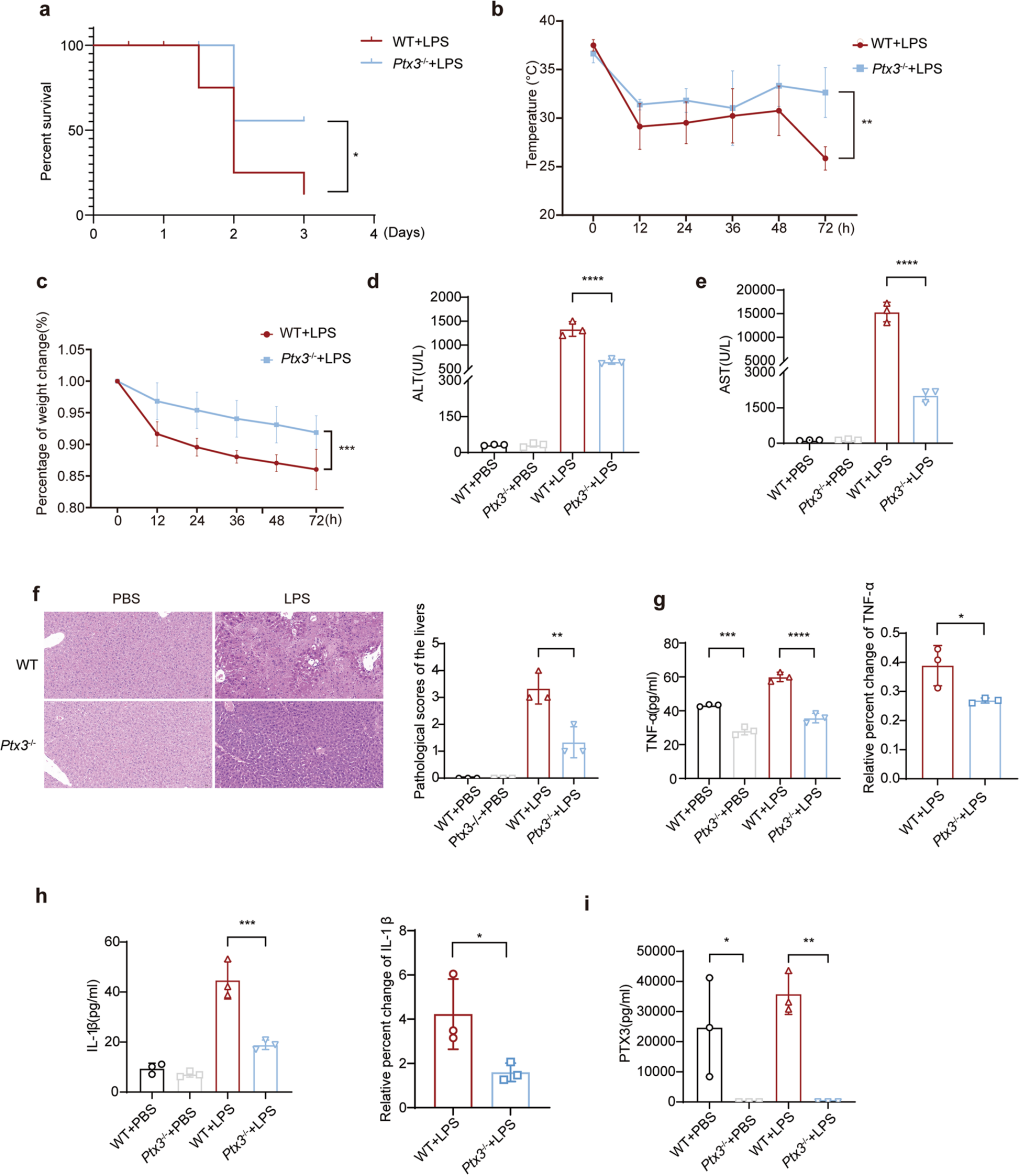
PTX3 deficiency alleviates LPS-induced liver injury in vivo. **a** Survival curve, (**b**) Rectal temperatures, and (**c**) Body weight of *Ptx3*^−/−^ and WT mice after injection with 10 mg/kg lipopolysaccharide (LPS) for 72 h (*n* = 8 mice per group). **d** Serum alanine aminotransferase (ALT) and (**e**) Aspartate aminotransferase (AST) levels, (**f**) Hematoxylin and eosin staining, (original magnification = 200×, scale bar = 100 μm) (**g**-**i**) TNF-α, IL-1β and PTX3 levels of *Ptx3*^−/−^ and WT mice in serum and liver tissue at 0 h and 72 h after injection with 10 mg/kg LPS (*n* = 3 mice per group). *P < 0.05, **P < 0.01, ***P < 0.001, ****P < 0.0001 by Student’s t-test or one-way ANOVA

To investigate the potential impact of PTX3 on inflammation induced by LPS, a global knockout mouse model of *Ptx3* (*Ptx3*^−/−^) was generated. We constructed an endotoxemia model by intraperitoneally injecting LPS (10 mg/kg) into *Ptx3*^−/−^ and wild-type (WT) mice. Survival curves revealed that the global deletion of *Ptx3* conferred protection against LPS-induced septic death, and the results revealed that the difference between the groups was statistically significant (*P* = 0.0397) (Fig. 1a), accompanied by less pronounced decreases in body temperature and weight (*n* = 6) (*P* = 0.0068 and *P* = 0.0002, respectively) (Fig. 1b and c). Alanine aminotransferase (ALT) and aspartate aminotransferase (AST) are important indicators of the degree of liver injury. Serum biochemical analysis revealed that *Ptx3* knockout led to a significant reduction in ALT and AST levels (*P* < 0.0001 and *P* < 0.0001, respectively) (Fig. 1d and e), indicating improved liver function in *Ptx3*^−/−^ mice compared with the other groups. Histological examination via H&E staining revealed more severe liver tissue damage in WT mice (*P* = 0.0015, Fig. 1f).

Serum PTX3 has been identified as a potential predictor of mortality during infection and is known to be associated with TNF-α and IL-1β, which are crucial proinflammatory mediators [24]. Therefore, we assessed the levels of serum cytokines, including PTX3, IL-1β and TNF-α, in mice 72 h after LPS treatment. The ELISA results revealed that the serum levels of proinflammatory factors such as TNF-α (*P* = 0.0002 and *P* < 0.0001, respectively) and IL-1β (*P* = 0.0002) were significantly lower in *Ptx3*^−/−^ mice than in WT mice (Fig. 1g and h), which was consistent with the trend observed for PTX3 levels (*P* = 0.0373 and *P* = 0.0050, respectively) (Fig. 1i). Moreover, the relative percentages of TNF-α and IL-1β producing cells in WT mice were significantly greater than those in *Ptx3*^−/−^ mice. Western blot analysis further confirmed the upregulation of IL-1β expression in the liver tissue of WT mice (Fig. S1). Collectively, these results indicate that *Ptx3* knockout attenuates inflammation induced by LPS in mice.

### PTX3 regulates LPS-induced hepatic ferroptosis

**Fig. 2:**
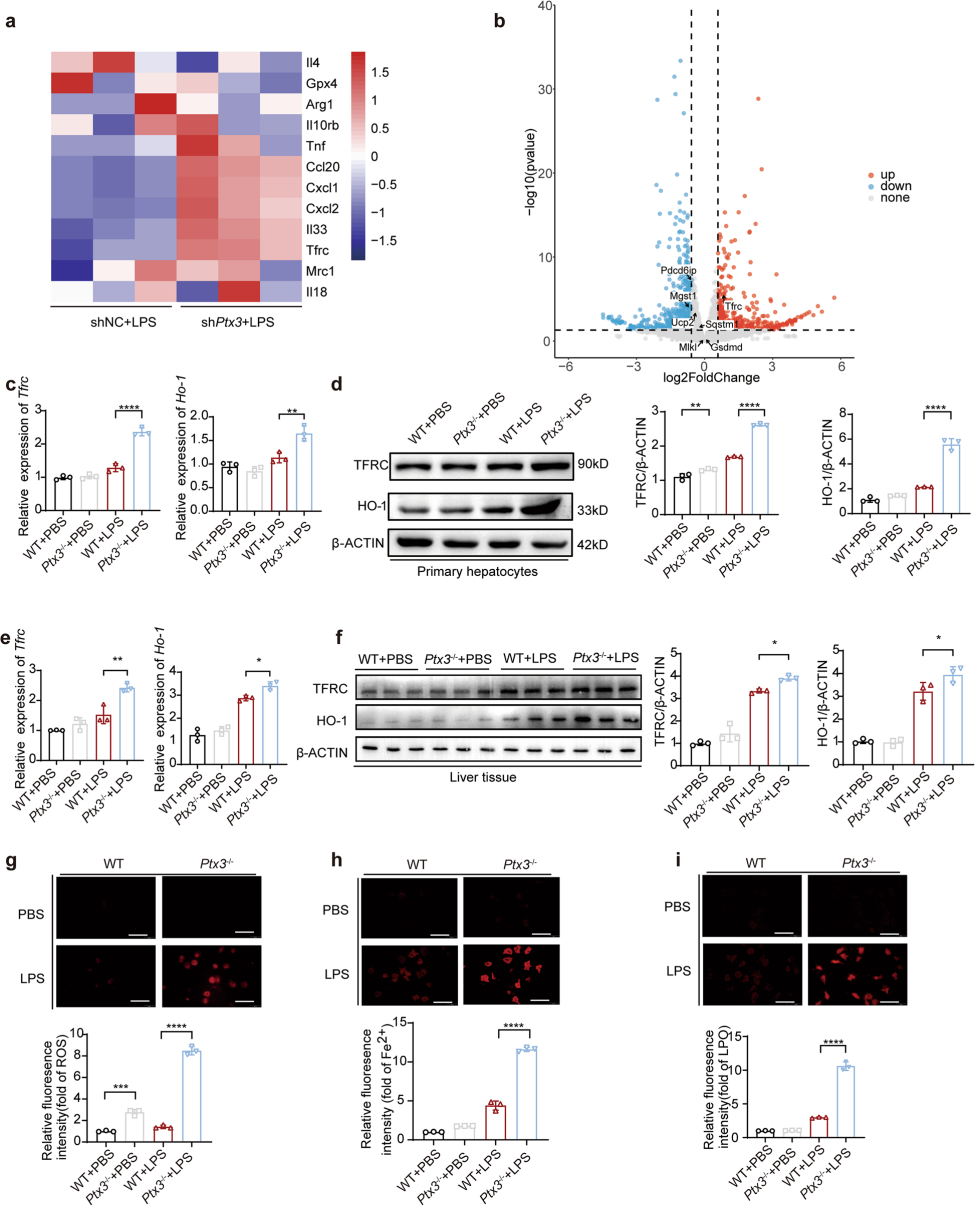
PTX3 inhibits hepatic ferroptosis. **a**, **b** Bulk mRNA sequencing data of mouse liver primary hepatocytes from WT and *Ptx3*^−/−^ after treated with 10 ng/mL LPS for 2 h (*n* = 3). **c** qRT-PCR and (**d**) Western blot detected the expression of TFRC and HO-1 in mouse liver primary hepatocytes from WT and *Ptx3*^−/−^ after treated with 10 ng/mL LPS for 12 h (*n* = 3). **e** qRT-PCR and **f** Western blot detected the expression of TFRC and HO-1 in *Ptx3*^−/−^ and WT mice after injection with 10 mg/kg LPS (*n* = 3 mice per group). **g** eactive oxygen species (ROS) levels in mouse liver primary hepatocytes measured by MitoSOX Dye Red with 10 ng/mL LPS for 12 h (*n* = 3). (original magnification = 200×, scale bar = 100 μm) (**h**) Intracellular Fe^2+^ levels measured by FerroOrange (red) with 10 ng/mL LPS for 24 h (*n* = 3). (original magnification = 200×, scale bar = 100 μm) (**i**) The levels of Lipid peroxide (red) in mouse liver primary hepatocytes after treated with 10 ng/mL LPS for 12 h (*n* = 3). (original magnification = 200×, scale bar = 100 μm) *P < 0.05, **P < 0.01, ***P < 0.001, ****P < 0.0001 by Student’s t-test or one-way ANOVA

LPS exposure increases hepatocyte apoptosis and necrosis, ultimately leading to liver damage. We first established stable *Ptx3*-knockdown (sh*Ptx3*) and control (shNC) AML12 cell lines and confirmed the *Ptx3*-knockdown efficiency via qRT‒PCR and fluorescence microscopy (Fig. S2a). We subsequently analyzed the effect of *Ptx3* on hepatocyte death via RNA sequencing in mouse AML12 cells with or without *Ptx3*-knockdown after treatment with LPS. A total of 383 differentially expressed genes (log_2_-fold change > 1 and padj < 0.05) were identified between the control group and the *Ptx3*-knockdown group. Screening for differentially expressed genes related to cell death revealed significant upregulation of *Tfrc* expression (Fig. 2a and b).

The increased expression of TFRC leads to cellular ferrous accumulation. The accumulation of labile iron augments HO-1 expression, and the upregulation of HO-1 regulates the expression levels of TFRC, causing a vicious cycle involving iron accumulation and TFRC and HO-1 upregulation [25–27]. We isolated the mouse primary hepatocytes from WT mice and *Ptx3*^−/−^ mice. qRT-PCR detected the efficiency (*P* < 0.0001, Fig S2c). Next, we assessed cell death in mouse primary hepatocytes obtained from WT mice and *Ptx3*^−/−^ mice under LPS treatment and observed increased *Tfrc* and *Ho-1* expression in the *Ptx3*^−/−^ mouse group (*P* < 0.0001 and *P* = 0.0035, respectively) (Fig. 2c), with no discernible differences in *Gsdmd*, *Slc40a1*, *Sqstm1*, *Mlkl*, *Lc3a* and *Lc3b* expression (Fig. S3a). Meanwhile, some biomarkers related with ferroptosis were detected by western blot (Fig. S3b). There was no difference between control and treatment group. Additionally, TFRC and HO-1 protein expression was elevated in *Ptx3*^−/−^ mouse primary hepatocytes (*P* < 0.0001 and *P* < 0.0001, respectively) (Fig. 2d), and similar results were confirmed in liver tissues from *Ptx3*^−/−^ mouse and WT mouse (Fig. 2e, *P* = 0.0021 and *P* = 0.0375, respectively) (Fig. 2f, *P* = 0.0423 and *P* = 0.0458, respectively). Both the western blot and qRT‒PCR analyses demonstrated that *Ptx3* deficiency significantly increased the expression of TFRC and HO-1, which were related to the intracellular iron content, confirming the correlation between PTX3 and ferroptosis.

Ferroptosis is characterized by intracellular iron accumulation, increased ROS levels and mis-controlled accumulation of lipid peroxidation (LPO), which are extremely harmful to cells. We treated primary hepatocytes with LPS and observed a significant increase in the level of the Fe^2+^ indicator FerroOrange in cells with the inhibition of *Ptx3*(*P* < 0.0001) (Fig. 2g). Additionally, ROS detection revealed that primary hepatocytes lacking *Ptx3* demonstrated increased levels of ROS (*P* < 0.0001) (Fig. 2h). We also examined the levels of LPO and confirmed that *Ptx3*-knockdown promoted the production of LPO (*P* < 0.0001) (Fig. 2i). In summary, these findings suggest that silencing *Ptx3* enhances the extent of LPS-induced ferroptosis by increasing the expression of TFRC.

### Inhibition of PTX3 promotes the migration of macrophages

**Fig. 3:**
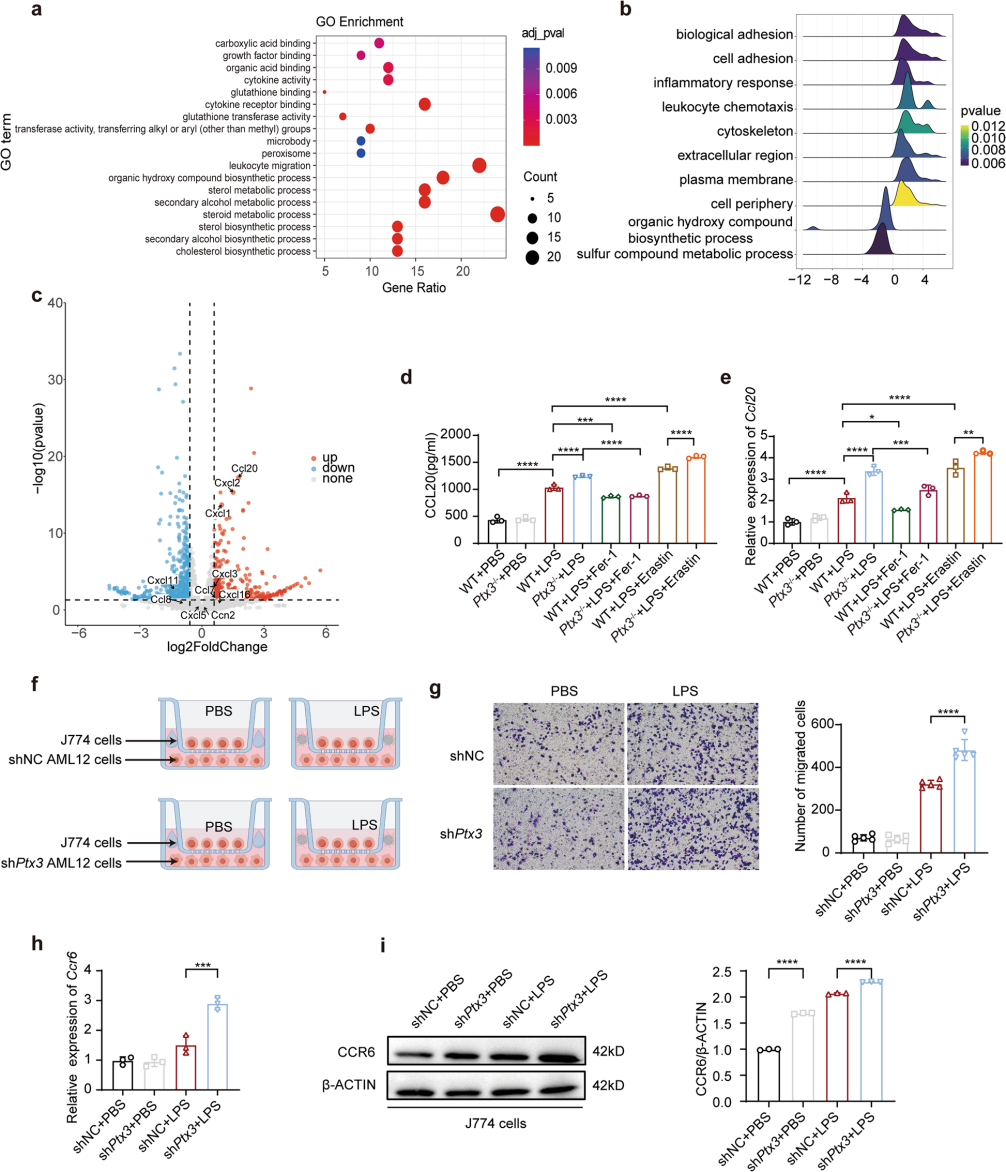
The absence of PTX3 promotes the migration of macrophage. **a** KEGG analysis, (**b**) GSEA analysis, (**c**) Volcano plot shown chemokines, (**d**) qRT-PCR, and (**e**) ELISA detected the expression of CCL20 in mouse liver primary hepatocytes from WT and *Ptx3*^−/−^ after treated with 10 ng/mL LPS for 12 h. And 10µM ferroptosis inhibitors Ferrostatin-1 for 12 h reduced CCL20 secretion. 20µM ferroptosis inducer Erastin increased the levels of CCL20 (*n* = 3). **f** The graph was the schematic of migration assays(By Figdraw.com, Copyright Code: YRURUef99f and IPYRY249b4). Migration of J774 cells co-cultured with mouse liver primary hepatocytes from WT and *Ptx3*^−/−^ was analyzed by Transwell assay after 48 h of coculture and stained with crystal violet (*n* = 5). (original magnification = 200×, scale bar = 100 μm) (**g**) qRT-PCR detected the expression of *Ccr6* in J774 cells cocultured with liver primary hepatocytes. **h** Western blot detected the expression of CCR5 and CCR6 in J774 cells cocultured with liver primary hepatocytes. *P < 0.05, **P < 0.01, ***P < 0.001, ****P < 0.0001 by Student’s t-test or one-way ANOVA

Kyoto Encyclopedia of Genes and Genomes (KEGG) and Gene Set Enrichment Analysis (GSEA) revealed enrichment of genes related to leukocyte migration and the leukocyte chemotaxis pathway (Fig. 3a and b). A volcano plot revealed that *Ccl20* was the most significantly upregulated chemokine in the knockdown group (Fig. 3c). CCL20, a kind of chemokine released by hepatocytes, has been reported to promote hepatocyte proliferation and macrophage activation [28]. To validate the sequencing results, we stimulated *Ptx3*^−/−^ and WT primary mouse hepatocytes with LPS and measured the expression of CCL20 in the cell pellets and medium. We observed that LPS significantly increased the expression of CCL20 in *Ptx3*^−/−^ primary mouse hepatocytes. We aimed to validate the link between ferroptosis and CCL20 secretion. The ferroptosis inducer erastin and the inhibitor ferrostatin-1 were added to the medium. Notably, after the suppression of *Ptx3*, compared with control hepatocytes, *Ptx3*^−/−^ primary mouse hepatocytes presented higher *Ccl20* levels in cell pellets and secreted more CCL20 into the cell supernatant after ferroptosis inducer stimulation. Correspondingly, the ferroptosis inhibitor led to the downregulation of CCL20 expression (Fig. 3d, *P* = 0.0033 and *P* = 0.0008, respectively) (Fig. 3e, *P* < 0.0001 and *P* = 0.0208, respectively). The conclusion was also demonstrated by AML12 cells with or without *Ptx3* knockdown (Fig S4 a and b).

To further verify whether CCL20 released by hepatocytes can promote the migration of macrophages, we conducted a transwell assay. *Ptx3*^−/−^ primary mouse hepatocytes were cultured in 6-well plates at a concentration of 4 × 10^5^ cells per well in the lower chamber. The primary hepatocytes were stimulated with 10 ng/mL LPS for 48 h. J774 macrophages were seeded in the upper chamber (Fig. 3f). Cell migration was observed via crystal violet staining. The results revealed a significant increase in the number of migrated cells in the group that was cocultured with *Ptx3*^−/−^ primary mouse hepatocytes (Fig. 3g) (*P* < 0.0001). Then we repeated this assay with Raw264.7 cell line and co-culturing with hepatocytes with or without *Ptx3* knockdown, the results were familiar with those of J774(Fig. S5c). As the sole receptor of CCL20, CCR6 is a type of G protein-coupled receptor widely expressed in various immune cells [29]. The CCL20/CCR6 axis also plays an important role in macrophage activation and polarization [30]. Next, the macrophages in the upper chambers were collected to measure the expression of CCR6. The results of the qRT‒PCR assay revealed that the secretion of CCL20 increased the RNA level of *Ccr6* in macrophages (Fig. 3h) (*P* = 0.0002). We quantified the RNA level of *Ccl20* in J774 cells and Raw cells in the presence and absence of LPS to further verify the result that increasing CCR6 expression of macrophages are influenced by the CCL20 secreted from hepatocytes. (Fig S4 c and d). And we tested the expression of CCR5 and CCR6 protein in J774 cells. The result of western blot revealed the *Ptx3*^−/−^ primary mouse hepatocytes stimulated by LPS improved the expression of CCR6 because of the secretion of CCL20(Fig. 3i) (*P* < 0.0001). Interestingly, the inhibition of *Ptx3* influenced CCR5 expression in the absence of LPS induced sepsis (Fig. S4 e). But there was no difference between control group and knockdown group under the condition of sepsis. Subsequently, we added the CCL20 recombinant proteins to the culture medium and detected the expression of *Ccr6* in macrophages. The result was also in consistent with previous results of co-culture (Fig. S4 f and g). Collectively, these results demonstrate that the inhibition of *Ptx3* can promote the migration of macrophages by CCL20/CCR6 axis.

### PTX3 can suppress M1 macrophage polarization

**Fig. 4:**
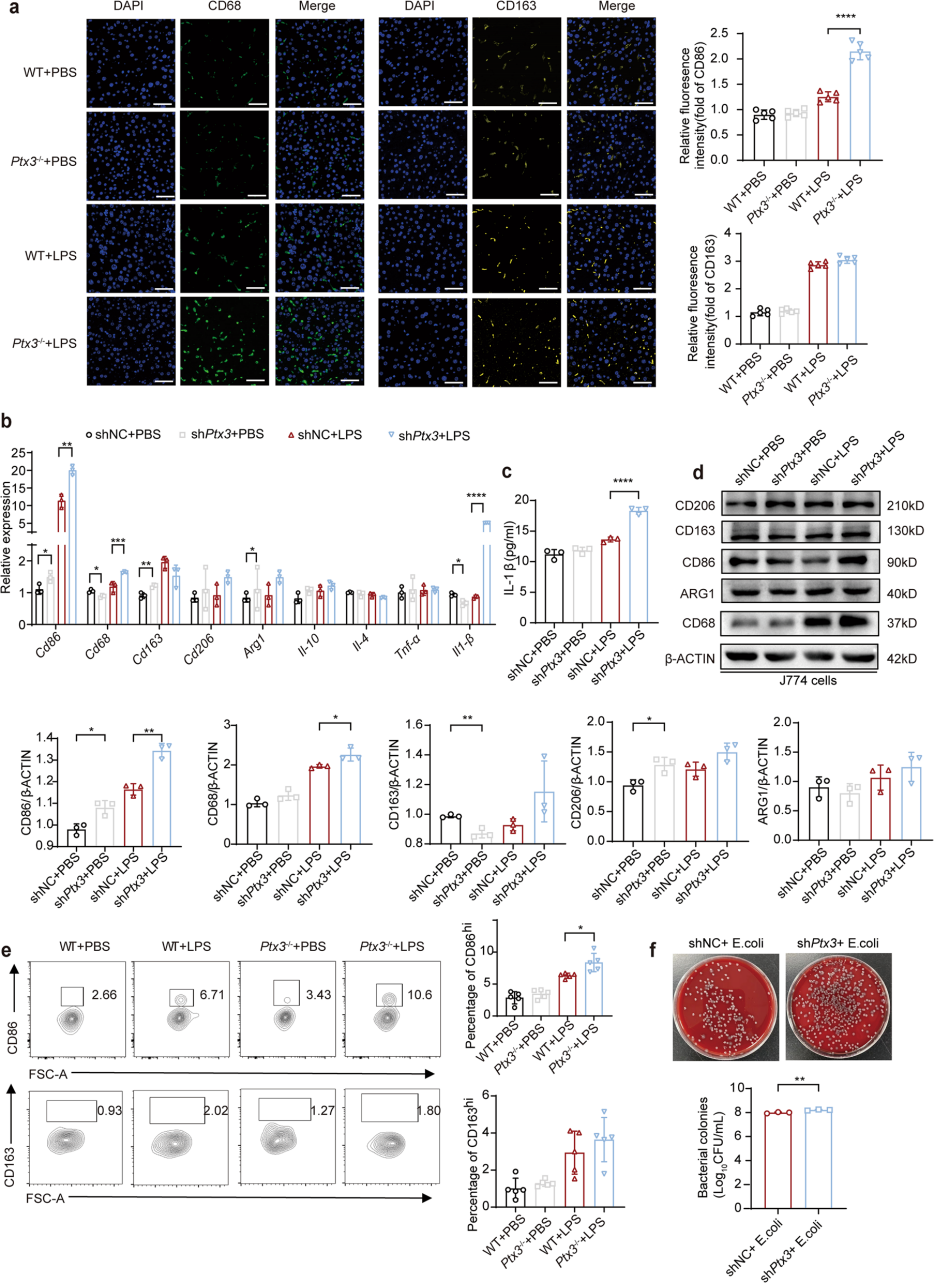
The absence of PTX3 regulates the polarization of M1 macrophage. **a** Immunofluorescence detected CD86 and CD163 in liver of *Ptx3*^−/−^ and WT mice after injection with 10 mg/kg LPS for 72 h and the statistical quantification of hepatic macrophages (*n* = 5). (original magnification = 400×, scale bar = 20 μm) (**b**) qRT-PCR detected the expression of M1 macrophage marker and M2 macrophage marker in J774 cells with or without PTX3 knockdown after treated with 10 ng/mL LPS for 24 h (*n* = 3). **c** ELISA detected the levels of IL-1β in medium supernatant of J774 cells with or without *Ptx3* knockdown after treated with 10 ng/mL LPS for 12 h (*n* = 3). **d** Western blot tested the expression of CD68, CD86, CD163, CD206 and ARG1 (*n* = 3). **e** Representative FACS plots and the statistical quantification of hepatic macrophages. **f** Phagocytic capacity detection of J774 cells with or without *Ptx3* knockdown by CFU assay. *P < 0.05, **P < 0.01, ***P < 0.001, ****P < 0.0001 by Student’s t-test or one-way ANOVA

Next, we investigated whether PTX3 has any effect on macrophage polarization. Immunofluorescence revealed increased expression of the M1 macrophage surface marker CD86 and the M2 macrophage surface marker CD163 in *Ptx3*^−/−^ mice with LPS for 72 h (Fig. 4a) (*P* < 0.0001). We knocked down *Ptx3* by using short hairpin RNA (shRNA) in the J774 cell line and assessed the efficiency of the knockdown by qRT‒PCR (Fig. S5a) and ELISA (Fig. S5b). We observed a significant reduction in PTX3 levels in the medium of J774 cells with *Ptx3* knockdown after treatment with LPS.

We subsequently validated the expression of the M1 macrophage markers *Cd68* (*P* = 0.0132), *Cd86* (*P* = 0.0028), and *Il-1β* (*p* < 0.0001), which were significantly increased in the knockdown group after 24 h of LPS stimulation, whereas there were no significant differences in the expression of the M2 macrophage markers *Arg1*, *Cd163*, *Cd206* and *Il-10*, as determined by qRT‒PCR (Fig. 4b). And there was no difference in the expression of *Tnf-α*. Interestingly, the qRT‒PCR and ELISA results revealed a significant increase in IL-1β levels after *Ptx3* knockdown in J774 cells, contrary to the results observed in the knockout mice (Fig. 4c) (*P* < 0.0001). IL-1β levels have been suggested to reflect phagocytosis by macrophages. The western blot results were the same as the qRT‒PCR results (Fig. 4d). To further assess the polarization of macrophages, flow cytometry analysis of the liver was performed. There was a significant difference in the expression of CD86 between *Ptx3*^−/−^ mice and WT mice treated with LPS (Fig. 4e) (*P* = 0.0152). A necessary process to resolve efficient inflammation is the phagocytosis of dying cells by macrophages [30]. We detected the phagocytosis of J774 macrophages. The number of *Escherichia coli* colonies on the blood plate was counted to determine the phagocytic capacity of the macrophages. The results of the macrophage phagocytosis assay revealed that the number of *Escherichia coli* colonies phagocytosed by *Ptx3*-knockdown macrophages was greater than that phagocytosed by control macrophages, which reflects the greater degree of phagocytosis in *Ptx3*-knockdown macrophages (Fig. 4f) (*P* = 0.0056).

Therefore, these results suggest that silencing *Ptx3* promotes the polarization of M1 macrophages and enhances the phagocytosis of macrophages to eliminate microorganisms.

### PTX3 modulates the NF-κB pathway to suppress macrophage polarization

**Fig. 5:**
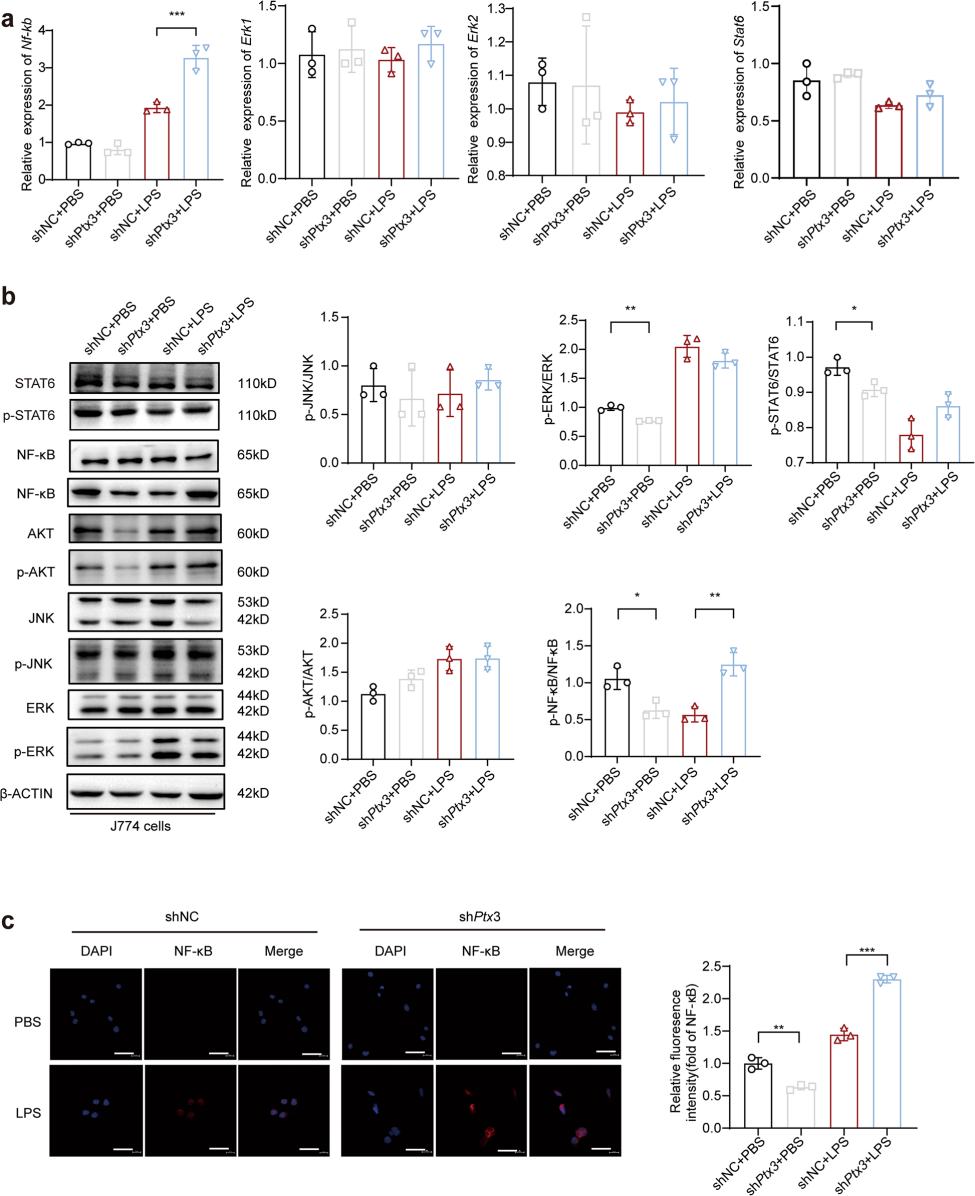
Effect of ERK, AKT, and NF-κB pathway mediated by PTX3 in J774 cells on polarization of M1 macrophage. **a** qRT-PCR measured the expression of *Nf-κb*,* Erk1*,* Erk2* and *Stat6* in J774 cells with or without *Ptx3* knockdown with 10 ng/mL LPS for 1 h (*n* = 3). **b** Western blot analyzed the levels of signaling pathway protein expression in J774 cells with or without *Ptx3* knockdown with 10ng/mL LPS for 4 h (*n* = 3). **c** Immunofluorescence staining detected the expression of NF-κB in J774 cells with or without *Ptx3* knockdown with 10ng/mL LPS for 4 h (*n* = 3). (original magnification = 400×, scale bar = 20 μm) *P < 0.05, **P < 0.01, ***P < 0.001, ****P < 0.0001 by Student’s t-test or one-way ANOVA

Signaling pathways play important roles in modulating the release of inflammatory mediators by LPS-stimulated macrophages. To explore the molecular mechanism of macrophage polarization, J774 cells with or without *Ptx3* knockdown were treated with LPS, and qRT‒PCR was used to assess the expression of *Nf-κb* (*P* = 0.0001), *Erk* and *Stat6* (Fig. 5a).

The results revealed that macrophages lacking *Ptx3* presented increased sensitivity to LPS stimulation, leading to significant upregulation of NF-κB expression after 1 h of LPS treatment. Additionally, western blot analysis demonstrated increased phosphorylation of components of the NF-κB pathway at 4 h poststimulation (*P* = 0.0187 and *P* = 0.0011, respectively) (Fig. 5b). These findings were further confirmed by immunofluorescence (Fig. 5c) (*P* = 0.0002). Hence, these results suggest that PTX3 inhibits M1 macrophage polarization by modulating the NF-κB pathway.

## Discussion

In this study, we demonstrated that PTX3 played a critical role in LPS-induced systemic inflammation and the influence of PTX3 on communication between parenchymal hepatic cells and immune cells is an important mechanism by which PTX3 promotes liver injury and septic mortality. We discovered that the knockout of *Ptx3* markedly ameliorated LPS-induced liver dysfunction and hepatocyte ferroptosis partially by upregulating the expression of *Tfrc* and *Ccl20*. Furthermore, we demonstrated that silencing *Ptx3* regulates macrophage polarization toward the M1 subtype by activating the NF-κB signaling pathway and recruiting these macrophages dependent on CCL20/CCR6 axis to the injured liver to phagocytose and clear harmful substances.

It has been well known that circulating increasing PTX3 levels are considered as an independent prognostic indicator in inflammatory diseases including COVID-19 and neuroinflammation [31, 32]. Xie’s study reported that administration of PTX3 provoked airway inflammation in asthma by promoting both eosinophils and neutrophils lung infiltration [33]. Anti-PTX3 antibodies was able to prevented lupus nephritis progression [34]. These findings suggest that PTX3 as candidate therapeutic agent for sepsis. Our study highlighted the pivotal role of PTX3 in the impact of interactions between cell death of hepatocytes and immune cells activation in sepsis.

Ferroptosis is a form of iron-dependent programmed cell death characterized by lipid peroxidation. An increasing number of studies have indicated that ferroptosis plays an important role in sepsis [35–37]. It is plausible that hepatocyte ferroptosis can mitigate inflammatory reactions, given the plethora of studies reporting that the production of inflammatory mediators during ferroptosis and that therapeutics targeting ferroptosis are proving to be effect for sepsis-induced organ damage [35, 38]. We observed that PTX3 was upregulated in the septic mouse model. The western blot and PCR results demonstrated that *Ptx3* suppressed the expression of TFRC in both the septic mouse model and the LPS-stimulated hepatocyte cells. Furthermore, *Ptx3* knockdown in hepatocytes increased the production of ROS and Fe^2+^. These findings indicate that *Ptx3* knockdown in hepatocytes suppresses liver injury progression by promoting ferroptosis. These findings are consistent with the findings of Alexis Kaushansky, who reported that lipid peroxides in ferroptotic cells lead to the elimination of liver-stage parasites [39]. The ferroptosis inducer erastin may also inhibit the NF-κB signaling pathway to attenuate the inflammatory response [39]. Therefore, the role of ferroptosis in inflammation may be a double-edged sword influenced by the tissue microenvironment. We hypothesized that ferroptotic hepatocytes protected other cells from infection by recruiting macrophages to the infected area to eliminate necrotic cells and pathogenic bacteria.

The liver plays a crucial role in the secretion of antisepsis agents, including cytokines and acute-phase proteins. Our data showed that *Ptx3* knockdown in hepatocytes induced ferroptosis and led to increased production of CCL20. CCL20 reportedly promotes M1-type polarization and macrophage recruitment by activating its specific receptor CCR6 [40–42]. Consistently, we found increased recruitment of M1-type monocyte-derived macrophages to an injury site via migration assays in vitro. Moreover, we determined that PTX3-regulated macrophage polarization in response to LPS infection was dependent on the NF-κB pathway.

Several studies have shown a close interaction between LPS-induced liver dysfunction through the NF-κB pathway [43, 44]. NF-κB is a key transcription factor that promotes the transcription of genes encoding proinflammatory cytokines and polarizes M2 macrophages into M1 macrophages [13]. Previous reports on PTX3-mediated regulation of the NF-κB pathway have yielded conflicting results. Koichi Node et al. demonstrated that PTX3 induced Akt phosphorylation, leading to a 35% reduction in nuclear NF-κB activation and a significant decrease in IL-1β and TNF-α levels [45]. In our study, we found that PTX3 modulated M1-type macrophage polarization. Specifically, *Ptx3* silencing regulated macrophage polarization toward the M1 subtype by activating NF-κB signaling pathways with a significant increase in IL-1β and TNF-α levels. However, we observed that the serum levels of IL-1β and TNF-α in *Ptx3* KO mice were lower than those in WT mice. We hypothesised that the contrary results of IL-1β and TNF-α in cellular and serum was due to the key role of phagocytosis of M1-type macrophages, which led to a decreased level of IL-1β and TNF-α released into the bloodstream by the harmful substances and the necrotic cells were cleared.

Our study has several limitations. The findings of our study are derived primarily from endotoxemia models established in mice, indicating PTX3 can be used as a potential predictive and therapeutic targe for sepsis. However, the safety and efficacy of PTX3 treatment was a challenge for us to study and also needed to be further investigated in more animal experiments and clinical trials. Additionally, our study focused on the role of PTX3 in LPS-induced liver injury, but the extent to which PTX3 contributes to defense against other infections, such as those caused by gram-positive bacteria and fungi, remains unclear.

**Fig. 6:**
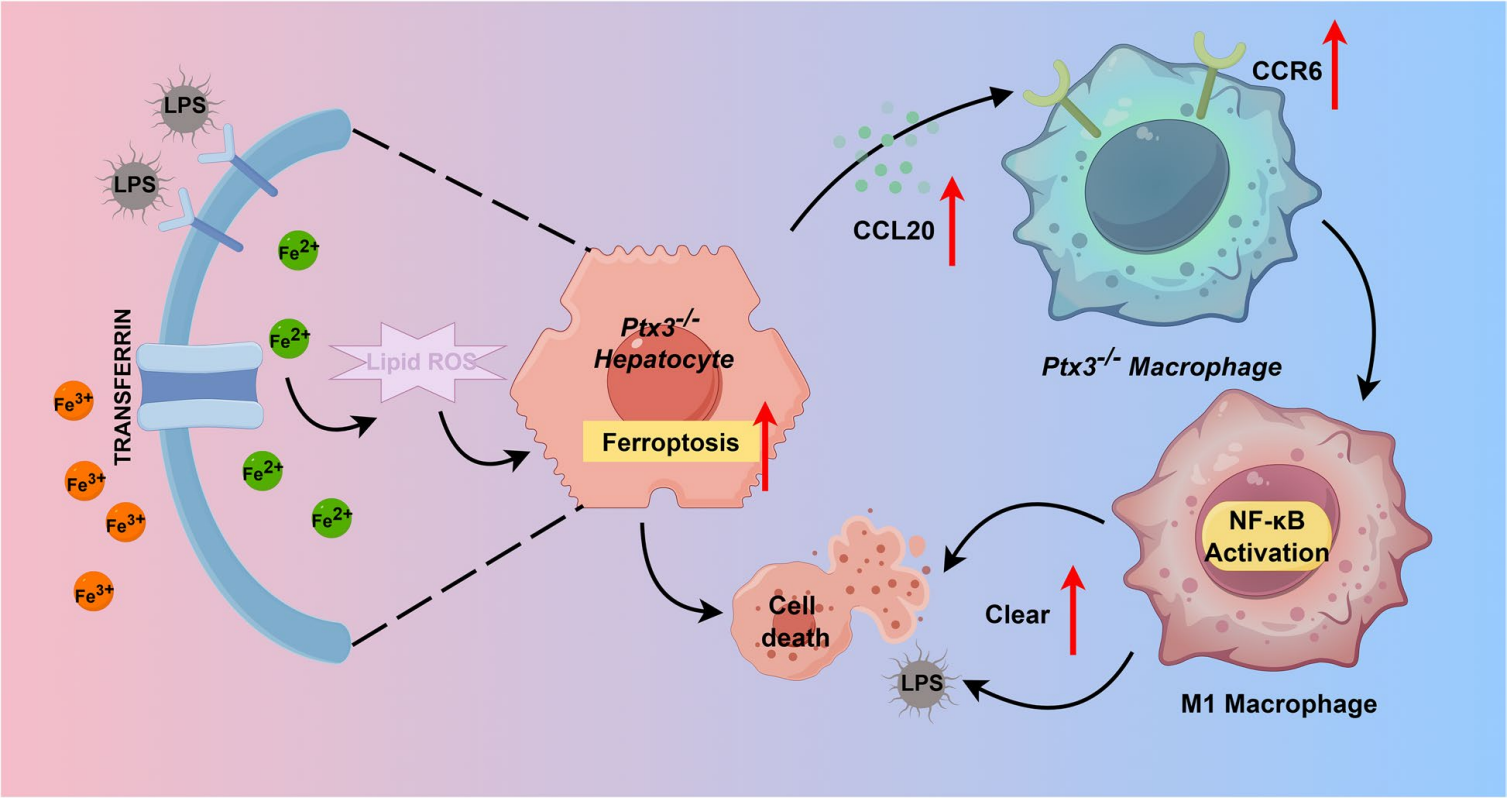
Schematic of the mechanism of PTX3 regulation of innate immunity and ferroptosis after LPS challenge. This graph showed that hepatocyte deficiency in PTX3 heightened *Tfrc* and *Ccl20* expression, thereby augmenting ferroptosis and attracting macrophages. Simultaneously, *Ptx3* deficiency in macrophages fostered M1 polarization via NF-κB pathway activation. These aggregated macrophages significantly contributed to the elimination of detrimental agents, facilitating inflammation clearance. (By Figdraw.com, Agreement ID: OSWSTd4d8d)

In summary, PTX3 inhibition can reduce LPS-induced liver injury through promoting hepatocyte ferroptosis and M1-type macrophage recruitment in sepsis (Fig. 6), which suggests that PTX3 might be a novel regulator involved in the treatment of bacterial infections.

## Methods

### Cell culture

AML12, J774, and RAW264.7 cells were procured from the Type Culture Collection of the Chinese Academy of Science (Shanghai, China) and cultured in DMEM (Gibco) supplemented with 10% fetal bovine serum (Gibco), 100 units/ml penicillin and 100 µg/ml streptomycin. All the cells were cultured at 37 °C with 5% CO_2_.

### Mice and treatment

The *Ptx3*-deficient mice were on the C57BL/6J background, generated as described previously [46], and obtained from the Shanghai Model Organisms Center. All the mice were fed under specific pathogen-free conditions at the Xiamen University Laboratory Animal Center, where they received a healthy diet and sterile water. The mice were utilized for follow-up experiments at 8 weeks of age. The mice were administered intraperitoneal injections of LPS (Sigma‒Aldrich, US). All efforts were made to minimize animal suffering, and the animal experiments were conducted in accordance with the guidelines approved by the Animal Ethics Committee of the Xiamen University Laboratory Animal Center (Approval number: XMULAC20230259).

### Western blot

The total protein samples were separated by 8% or 10% SDS‒polyacrylamide gel electrophoresis, immediately transferred onto 0.45-mm PVDF membranes (Roche, 03010040001), blocked in TBST (Tris-buffered saline with Tween 20) containing 5% nonfat dried milk at room temperature for 2 h, and probed with primary antibodies (4 °C overnight). Following incubation, the membranes were extensively washed with TBST. Afterward, the blots were subsequently incubated with the appropriate secondary antibodies (1:5000, CST, #7074) at room temperature for 1.5 h. After incubation, the membranes were washed in TBST. Finally, the protein bands were visualized via chemiluminescence using an enhanced chemiluminescence (ECL) substrate. The relative signal intensity of the detected protein was normalized to that of β-actin or GAPDH and quantified with ImageJ. The antibodies used for Western blotting included antibodies against TFRC(1:1000, Abcam, ab214039), HO-1(1:1000, Abcam, ab305290), NRF2(1:1000, CST, #12721), GPX4(1:1000, CST, #52455), SLC7A11(1:1000, CST, #98051), CCR5(1:1000, Abcam, ab7346), CD163(1:1000, Abcam, ab182422), CD206(1:1000, Abcam, ab64693), CD86(1:1000, Abcam, ab239075), CD68(1:1000, Abcam, ab283654), Arginase-1(1:1000, CST, #93668), NF-κB (1:1000, Abcam, ab32536), phospho-NF-κB (1:1000, CST, #3033), ERK1/2 (1:2000, Abcam, ab184699), phospho-ERK1/2 (1:1000, Abcam, ab81298), AKT (1:1000, Abcam, ab38449), phospho-AKT (1:2000, CST, #4060), JNK (1:1000, CST, #9252), phospho-JNK (1:2000, CST, #9251), STAT6 (1:1000, CST, #9362), phospho-STAT6 (1:1000, CST, #9361T), β-actin (1:2500, Abcam) and GAPDH (1:2500, Abcam).

### Primary mouse hepatocyte isolation

Primary hepatocytes from *Ptx3*^−/−^ and WT mice were isolated via the perfusion method. In brief, the mice were deeply anesthetized with isoflurane. The liver was filled with Ca^2+^-free EBSS solution, followed by collagenase solution (0.25 mg/mL, Sigma‒Aldrich) at 37 °C. Then, the gallbladder and excess tissue were immediately removed, and the liver was ground. The cell suspension was passed through a 70 μm cell strainer, and hepatocytes were obtained after purification by 50% Percoll (Sigma‒Aldrich) gradient centrifugation for approximately 5 min.

### Enzyme-linked immunosorbent assay (ELISA)

The levels of serum TNF-α, IL-6 and IL-1β in each group, as well as the medium supernatant levels of IL-1β and CCL20, were detected via ELISA kits (Multi Sciences, China). The level of PTX3 in the serum and medium supernatant was measured with an ELISA kit (Life Sciences, Solarbio, China).

### Quantitative real-time PCR assay

 Total RNA was extracted via an RNA extraction kit (ES Science) according to the manufacturer’s instructions. cDNA was synthesized via HiScript^®^III RT SuperMix for qPCR with gDNA wiper (EnzyArtisan, Cat. R202) subjected to real-time PCR via the Universal SYBR qPCR Mix (EnzyArtisan, Cat. Q204). The relative RNA levels were calculated via the comparative (∆∆Ct) method. The primer sequences are listed in Table S1.

### Lipid peroxidation assay

Lipid peroxidation was measured by BODIPY 581/591 C11 (S0043S, Beyotime). The cells were seeded in 6-well plates and incubated overnight. After treatment with 10 ng/ml LPS for 12 h, the medium was removed. The cells were subsequently washed twice with PBS. Afterward, 400 µL of PBS containing C11-BODIPY (5 µM) was added, and the mixture was incubated for 30 min at 37 °C in the dark. The cells were promptly observed under a fluorescence microscope (Leica Biosystems, Wetzlar, Germany; DMi8). The relative fluorescence intensity of LPO was quantified via ImageJ software.

### Immunofluorescence staining

For the immunofluorescence staining, AML12 cells were cultured in 12-well plates with a cover glass in each well. Following LPS treatment, the cells were immediately washed with PBS and then fixed with 4% paraformaldehyde for 15 min. The samples were subsequently blocked with 5% BSA containing 0.2% Triton X-100 for 1 h in the dark and incubated overnight at 4 °C with primary antibodies. After washing, the samples were incubated for 1.5 h with the secondary antibody. Images were captured via an inverted fluorescence microscope (Leica Biosystems, DMi8).

### Histological analysis of mouse livers

Liver tissues were directly fixed in freshly prepared 4% paraformaldehyde fixative solution after dissection. These samples were incubated overnight at 4 °C in 100 mM sodium phosphate buffer (pH 7.4) containing 30% sucrose and then embedded in paraffin wax at 56 °C. These samples were finally cut into 5 μm-thick slices and stained with hematoxylin and eosin (H&E) for histopathological analysis.

### Mitochondrial reactive oxygen species (mROS) measurement

mROS were measured via the MitoSOX™ Red mitochondrial superoxide indicator (YEASEN, Shanghai, China) according to the manufacturer’s protocols. AML12 cells were seeded onto cover slips in 12-well plates and treated with LPS for 12 h. The cells in all the groups were incubated with 5 µM MitoSOX™ reagent working solution for 30 min at 37 °C in the dark. After the samples were washed three times with warm PBS, the intensity of the ROS was assessed via fluorescence microscopy.

### Measurement of intracellular Fe2+

The FerroOrange fluorescent probe Kit was used according to the manufacturer’s protocol for the measurement of intracellular Fe^2+^. Briefly, AML12 cells were cultured in AML12-conditioned medium in 12-well plates. The cells were subsequently rinsed and incubated with 1 µM FerroOrange for approximately 30 min. The cells were promptly observed under a fluorescence microscope (Leica Biosystems, Wetzlar, Germany; DMi8). The relative fluorescence intensity of Fe^2+^ was quantified via ImageJ software.

### RNA-sequencing analysis

RNA was extracted from the liver tissues. The cDNA was sonicated via the Covaris S2 ultrasonicator, and the libraries were prepared via the KAPA High-Throughput Library Preparation Kit. The BGISEQ-500 sequencing platform was used to sequence all the libraries. The levels of RNA expression were examined via the fragments per kilobase of transcript per million mapped reads method. Genes that were differentially expressed after castration were identified via the fold-change method with the R package DEGseq. Genes with a log_2_ fold change > 1 and padj < 0.05 were deemed to be significantly differentially expressed.

### Serum ALT and AST measurement

The serum ALT and AST levels were detected via assay kits (Nanjing Jiancheng Bioengineering Institute, China). All quantifications were performed in strict accordance with the manufacturer’s protocols. The whole blood of the mice was stored at room temperature for 2 h and subsequently centrifuged for 10 min at 1000 × g to collect the serum.

### Lentivirus-mediated shRNA transfection

The silencing of *Ptx3* was achieved via the transduction of AML12 cells and J774 cells via lentiviral plasmids (LV3-H1/GFP&Puro) encoding *Ptx3* shRNA (LV-*Ptx3*-RNAi) or negative control shRNA (hU6-MCS-CBh-gcGFP-IRES-puromycin). The lentiviral plasmids were targeted eGFP-tagged protein to determine cellular expression. Only live cells that have been successfully transfected will be detected green fluorescence. Puromycin was used for the selection of stable colonies (Genepharma, Shanghai).

### Migration assays

Six-well Matrigel-coated chambers with 8-µm pore membranes (Corning Life Sciences, cat. no. 353097) were used to measure the migration capacity of the cells. RAW264.7 cells or J774 cells (1 × 10^6^/well) were placed in the upper chamber. Primary hepatocytes were cultured in 6-well plates at a concentration of 4 × 10^5^ cells per well in the lower chamber. The primary hepatocytes were stimulated with 10 ng/mL LPS for 48 h. The cells in the chambers were cultured in 5% CO_2_ at 37 °C. Then, the cells that migrated were fixed with 4% paraformaldehyde and stained with crystal violet (0.1%). The number of migratory cells was tested via ImageJ analysis software (Media Cybernetics, Bethesda, MA, USA). The cells that migrated were observed by the fluorescence microscopy. And the results were quantified by counting the number of migrated cells in the same part and expressed as standardized values for three separate experiments, with each assay counting 5 fields.

### Flow cytometry

The livers were removed from the dissected mice, placed in staining buffer (2% fetal bovine serum and 0.2% penicillin/streptomycin in 1× PBS solution) and ground through a strainer to collect single-cell suspensions in centrifuge tubes. Laminar propria immune cells were isolated with RPMI 1640 containing 200 U/ml collagenase VIII (Sigma Aldrich) and 0.15 mg/ml DNase I (Roche) for 30 min at 37 °C with shaking. Lymphocytes were enriched at the interface between a gradient of 40% and 70% Percoll (Cytiva). Single-cell suspensions were stained with a combination of fluorescently labeled monoclonal antibodies. For the cell surface molecules, the following antibodies were used for staining: Fixable Viability-eFluor780, CD45.2-APC (109814, Biolegend), CD11b-Brilliant Violet 711 (101242, Biolegend), F4/80-PE (565410, BD), CD86-Brilliant Violet 650 (564200, BD) and CD163-Super Bright 600 (2653085, Invitrogen). Data were acquired with a Cytoflex LX (Beckman Coulter) multicolor flow cytometer. All the flow cytometric data were analyzed and plotted via FlowJo software (TreeStar, Ashland, OR, USA).

### Macrophage phagocytosis assay

J774 cells were cultured in 6-well plates (1 × 10^6^/well) and washed 3 times with PBS, and *Escherichia coli* strain suspensions (MOI of 20:1) were added to the cell culture medium. Infected J774 cells were incubated with lysozyme (at a concentration of 20 µM; Sigma‒Aldrich, China) at 37 °C for 1.5 h. After 0 h (control group) and 24 h, the J774 cells were washed 3 times with PBS and lysed with a mixture of 0.25% trypsin and 0.025% Triton X-100 (Solarbio, China). The cell lysate (20 µl) was used for the CFU assay, and CFUs were determined via TSA plate counting: the lysate was diluted 100-fold in PBS and then incubated with TSA at 37 °C for 24 h.

### Statistical analysis

All the data are expressed as the means ± standard deviations. Statistical analyses were performed with GraphPad Prism (GraphPad, San Diego, CA). Student’s *t* test was used to compare the differences between groups. One-way analysis of variance (ANOVA) was used for comparisons among different groups. A *P* value of less than 0.05 was considered significant.

## Supplementary Information

Supplementary Material 1.

## Data Availability

All data associated with this study are present in the paper or the Supplementary Materials.

## Abbreviations

PTX3: Pentraxin 3
LPS: Lipopolysaccharide
ROS: Reactive oxygen species
TFRC: Transferrin receptor
TNF-α: Tumor necrosis factor-α
IL-1β: Interleukin-1 beta
WT: Wild-type
ALT: Alanine aminotransferase
AST: Aspartate aminotransferase
ELISA: Enzyme-linked immunosorbent assay
shPtx3: Ptx3-knockdown
LPO: Lipid peroxidation
KEGG: Kyoto Encyclopedia of Genes and Genomes
GSEA: Gene Set Enrichment Analysis
TBST: Tris-buffered saline with Tween 20
PAGE: Polyacrylamide gel electrophoresis
mROS: mitochondrial reactive oxygen species
