# Supplementary material for: Pentraxin-3 modulates hepatocyte ferroptosis and the innate immune response in LPS-induced liver injury
Source: Mol Biomed. 2024 Dec 12;5:68. doi: 10.1186/s43556-024-00227-6 (PMC11638432; doi:10.1186/s43556-024-00227-6)
Supplement: Supplementary file 1 — Supplementary Material 1. [file 43556_2024_227_MOESM1_ESM.pdf]

Fig. S1. Western blot analysis expression of IL1β in PTX3-ko and WT mice at 0h and 72h after injection with 10 mg/kg LPS (n = 5 mice per group).

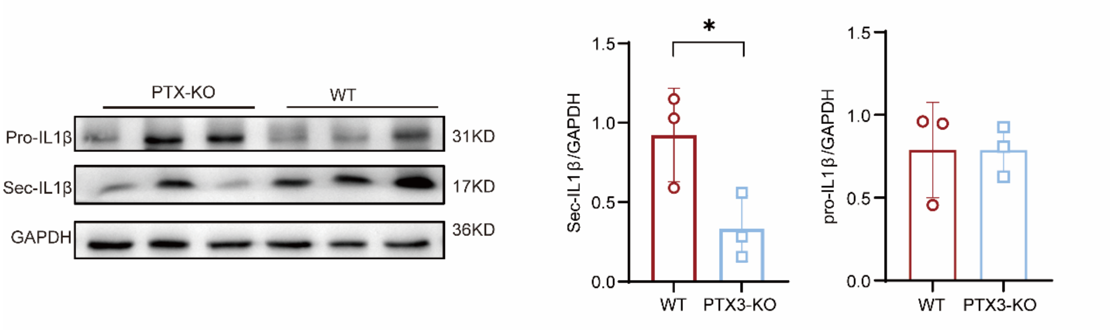

Fig. S2.

(a) Fluorescence microscopy confirmed the efficiency of PTX3 in AML12 cell lines. (original magnification = ×200, scale bar = 100 μ m)

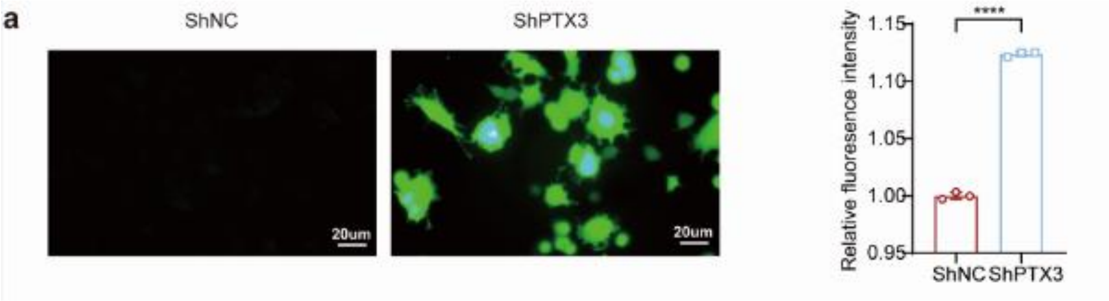

(b,c)qRT-PCR tested the efficiency of PTX3 in AML12 cell lines and hepatocytes isolated from mice(n=3).

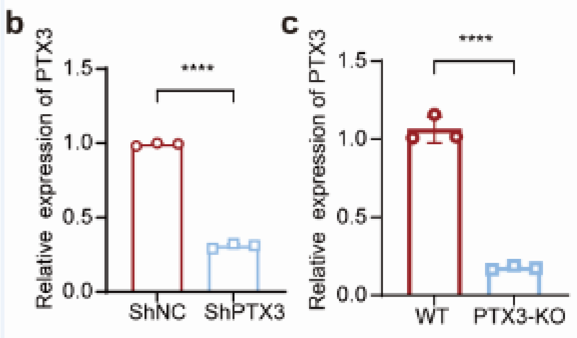

qRT-PCR detected the expression of TFRC(d) and HO-1(e) in mouse AML12 cells with or without PTX3 knock down after treated with 10 ng/mL LPS for 12h (n=3).

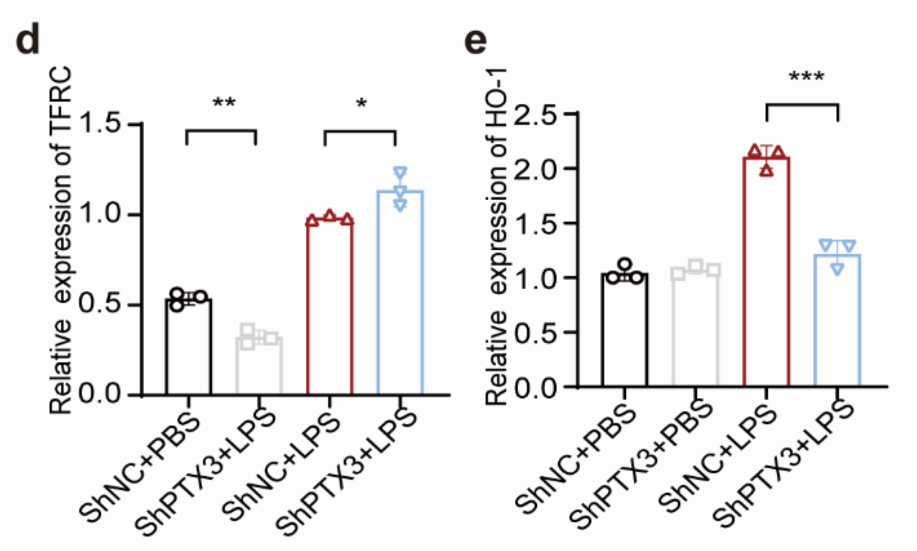

(f) The levels of Lipid peroxide (red) in mouse liver primary hepatocytes after treated with 10 ng/mL LPS for 12h (n=3). (original magnification = ×400, scale bar = 20μm)

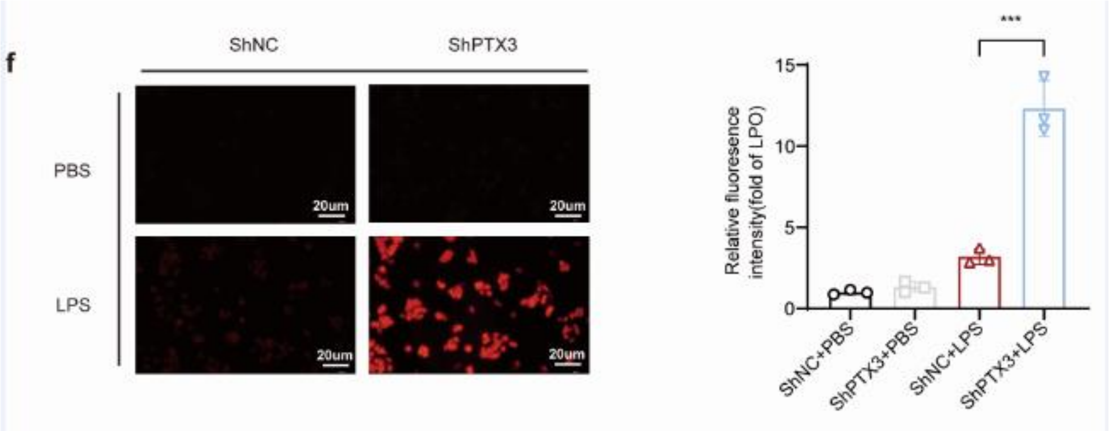

(g) Intracellular Fe2+ levels measured by FerroOrange (red) with 10 ng/mL LPS for 24h (n=3). (original magnification = ×200, scale bar = 100μm)

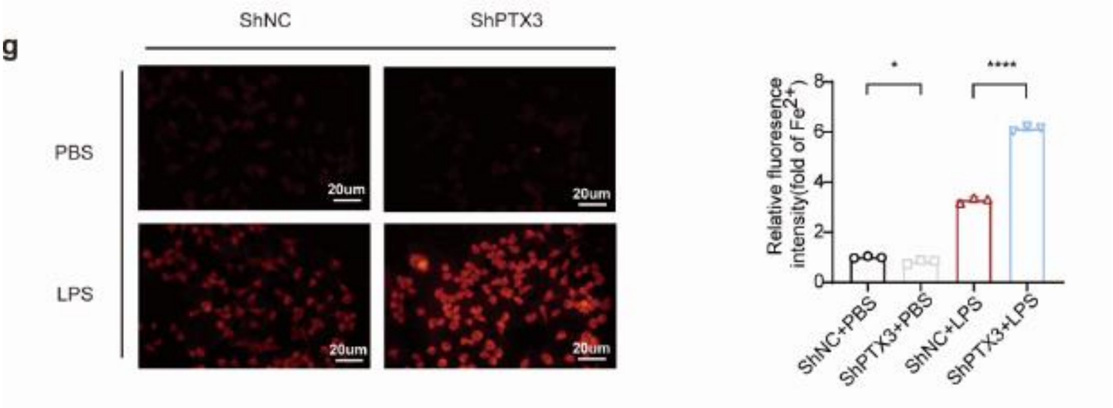

(h) ROS levels in mouse liver primary hepatocytes measured by MitoSOX Dye Red with 10 ng/mL LPS for 12h (n=3). (original magnification = ×200, scale bar = 100μm)

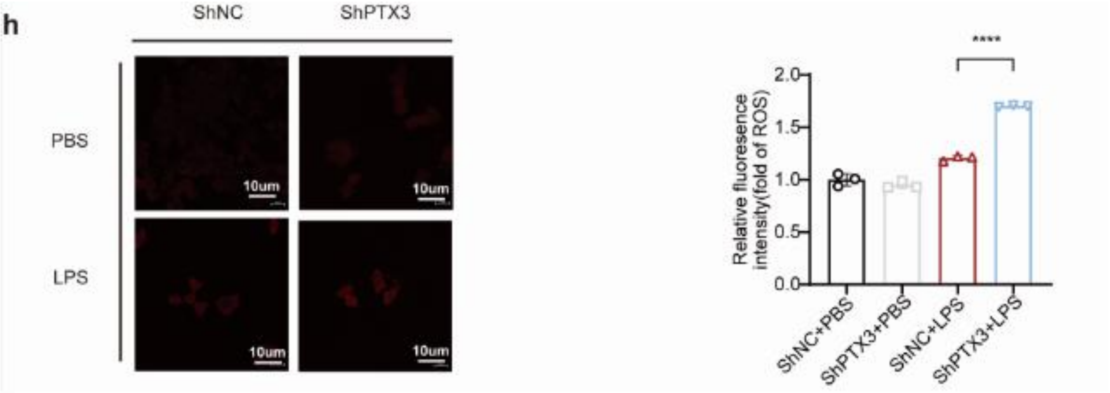

Fig. S3. qRT-PCR (a) and western blot (b) detected the expression of cell death models in AML12 cells with or without PTX3 knockout after treated with 10 ng/mL LPS for 12h.

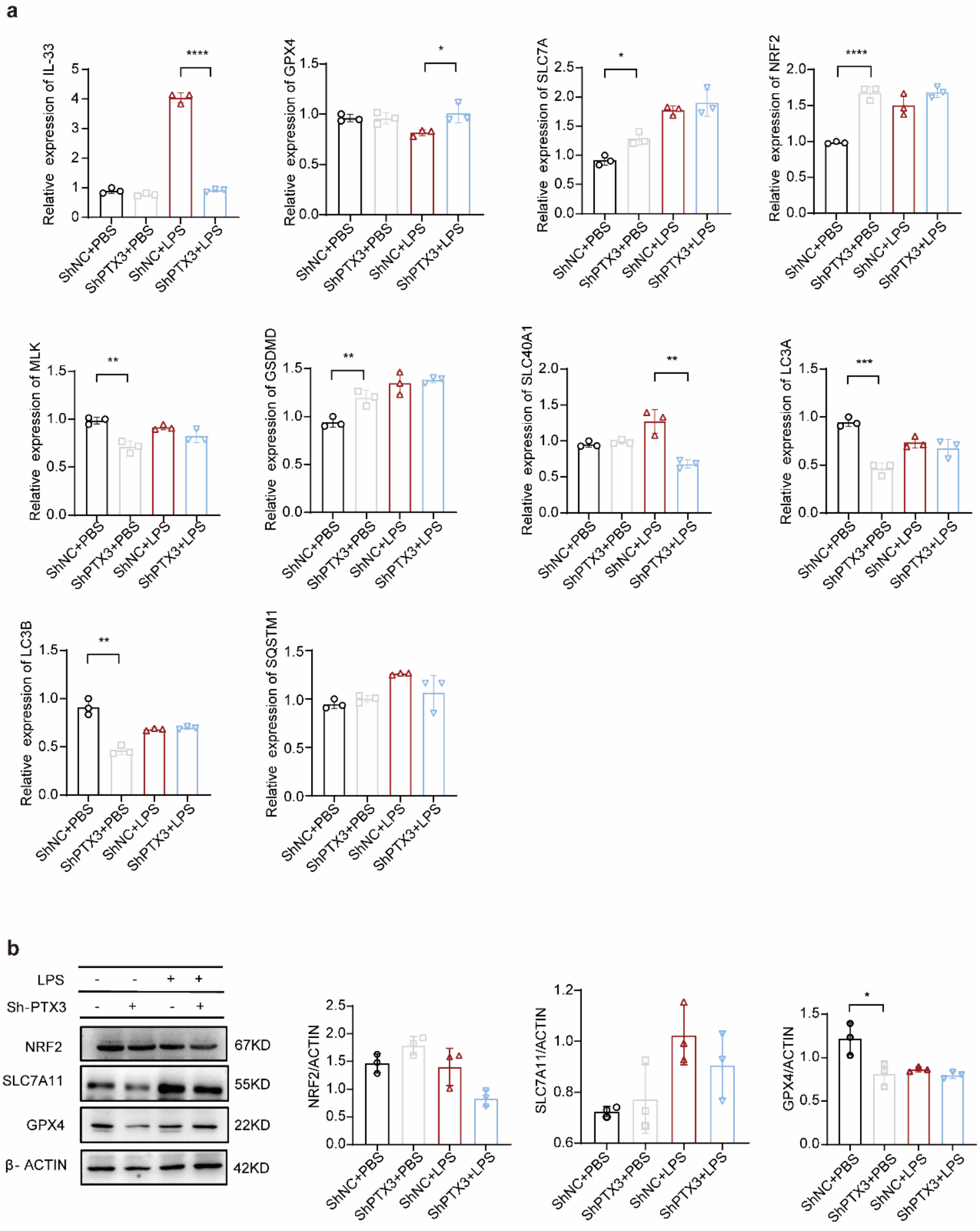

Fig. S4. qRT-PCR(a) and ELISA(b) detected the expression of CCL20 in mouse liver primary hepatocytes from WT and PTX3-/- after treated with 10 ng/mL LPS for 12h. And 10μM ferroptosis inhibitors Ferrostatin-1 for 12h reduced CCL20 secretion. 20μM ferroptosis inducer Erastin increased the levels of CCL20 (n=3).

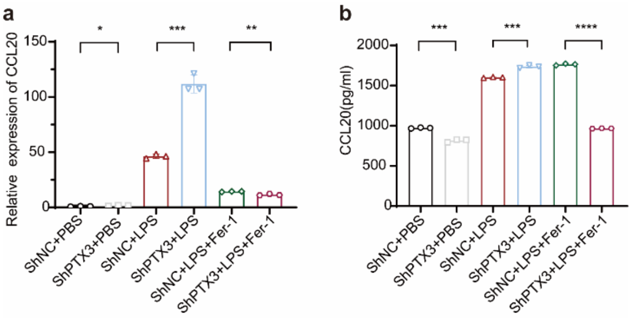

(c,d) qRT-PCR detected the expression of *Ccl20* in J774 cells and Raw264.7 cells after treated with 10 ng/mL LPS.

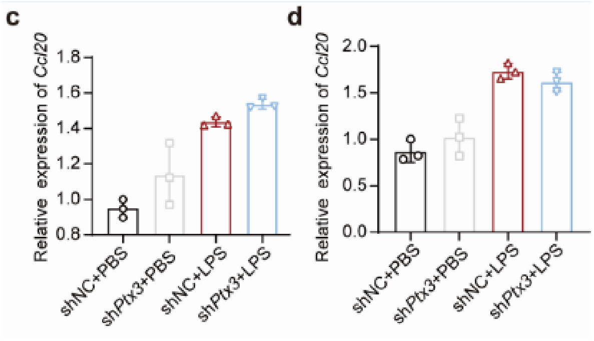

(e) western blot detected the expression of CCR5 in J774 cells with or without *Ptx3* knockdown after treated with LPS.

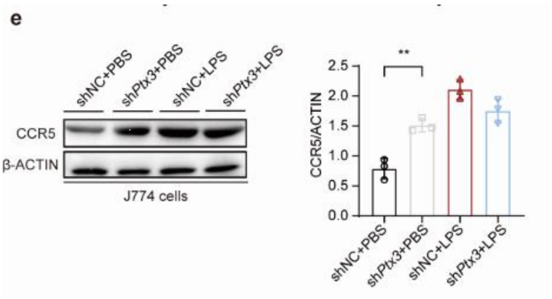

(f,g) qRT-PCR detected the expression of *Ccr6* in J774 cells and Raw 264.7 cells with or without *Ptx3* knockdown after treated with CCL20 recombinant proteins.

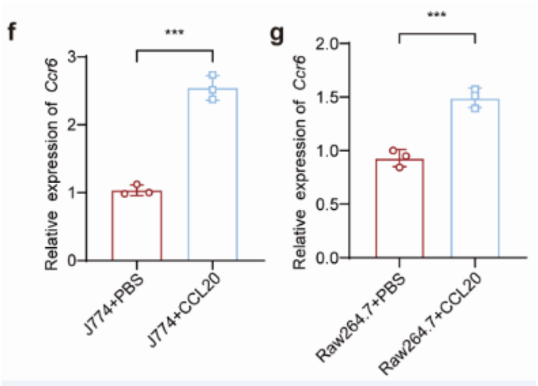

Fig. S5. qRT-PCR(a) and Elisa (b) tested the efficiency of knock down PTX3 (n=3). Cell migration was tested via crystal violet staining(c) (n=5). (original magnification = 200×, scale bar = 100μm)

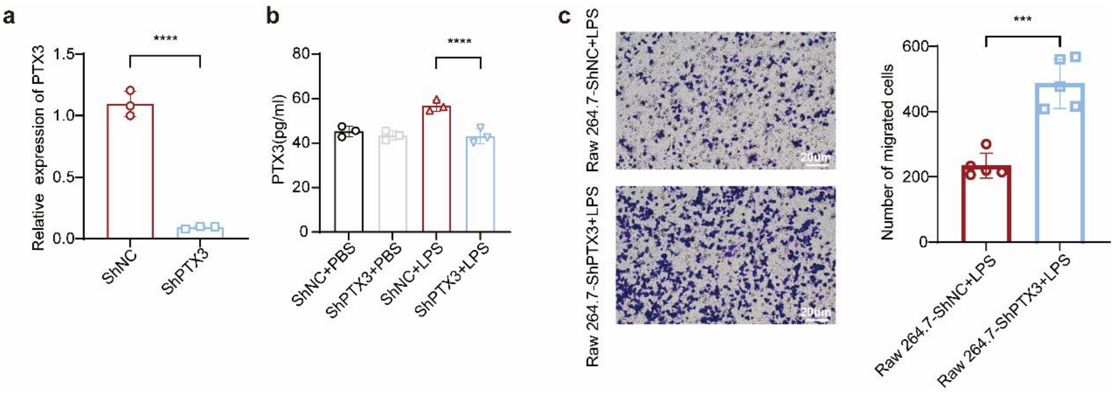

Table S1

Gene	Species	Forward primer	Reverse primer
CCL20	Mice	GTGGGTTTCACAAGACAGATGGC	CCAGTTCTGCTTTGGATCAGCG
PTX3	Mice	AAGTGGAACCCCTATGAGATTCAG	CCAGCTTGTTCTCCTTTCCACC
GAPDH	Mice	CATCACTGCCACCCAGAAGACTG	ATGCCAGTGAGCTTCCCGTTCAG
IL-33	Mice	CTACTGCATGAGACTCCGTTCTG	AGAATCCCGTGGATAGGCAGAG
TNF-α	Mice	GGGTTGTACCTTGTCTACTCCCAG	GAGATAGCAAATCGGCTGACG
GSDMD	Mice	GCGCTTTGTTCCATCGGAAAG	CCATTTCCAAGCTCTCCAGTTCTG
MLKL	Mice	GAATACCGTTTCAGATGTCAGCC	CTTCCACGCTAATTTGCAACTG
TFRC	Mice	GAAGTCCAGTGTGGGAACAGGT	CAACCACTCAGTGGCACCAACA
IL-1β	Mice	TGGACCTTCCAGGATGAGGACA	GTTCATCTCGGAGCCTGTAGTG
Slc7a11/xCT	Mice	CTTTGTTGCCCTCTCCTGCTTC	CAGAGGAGTGTGCTTGTGGACA
Arg1	Mice	AAGAATGGAAGAGTCAGTGTGG	GGGAGTGTTGATGTCAGTGTG
CD68	Mice	GCTTCTGCTGTGGAAATGCAAG	TGAGCAGCCTGTAGCCTTAGAGA
CD86	Mice	ACGTATTGGAAGGAGATTACAGCT	TCTGTCAGCGTTACTATCCCGC
Gpx4	Mice	CCGGCTACAACGTCAAGTTTG	CCCTTGGGCTGGACTTTCATC
Nrf2/Nfe2l2	Mice	TTCCCATTTGTAGATGACCATGAG	CTCCATGTCCTGCTCTATGCTG
HO-1/Hmox1	Mice	CACTCTGGAGATGACACCTGAG	GTGTTCCTCTGTCAGCATCACC
FPN1/Slc40a1	Mice	CCATAGTCTCTGTCAGCCTGCT	CTTGCAGCAACTGTGTCACCGT
LC3A/Map1lc3a	Mice	CGTCCTGGACAAGACCAAGT	ACCATCTACAGGAAGCCGTC
LC3B/Map1lc3b	Mice	CATGTTAACATGAGCGAGTTGGTC	GTTCATAGATGTCAGCGATGGG
NF-κB	Mice	GCTGCCAAAGAAGGACACGACA	GGCAGGCTATTGCTCATCACAG
SQSTM1	Mice	GCTCTTCGGAAGTCAGCAAACC	GCAGTTTCCCGACTCCATCTGT
ERK1	Mice	GGCTTTCTGACGGAGTATGTGG	GTTGGAGAGCATCTCAGCCAGA
ERK2	Mice	TCAAGCCTTCCAACCTCCTGCT	AGCTCTGTACCAACGTGTGGCT
Stat6	Mice	AGATCTTCAACGACAACAGCCTC	CCAGGACACCATCAAACCACTG
CCR6	Mice	ATGCGGTCAACTTTAACTGTGG	CCCGGAAAGATTTGGTTGCCT
IL-4	Mice	GAACTCTAGTGTTCTCATGGAGCTG	TCTTTCAGTGATGTGGACTTGGAC
CD163	Mice	TGTTCAGGAAGATTGGAAGTGAG	CCTCACTGGCATTAACTCGACC
CD206	Mice	GTTCACCTGGAGTGATGGTTCTC	AGGACATGCCAGGGTCACCTTT
IL-10	Mice	CGGGAAGACAATAACTGCACCC	CGGTTAGCAGTATGTTGTCCAGC

Fig.4 d

CD206

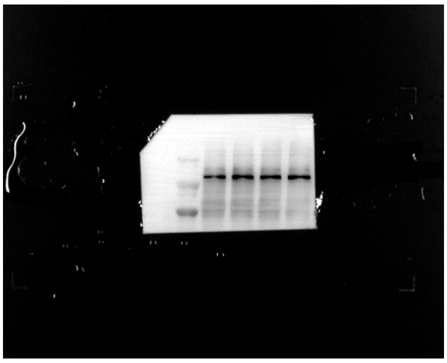

210KD

CD86

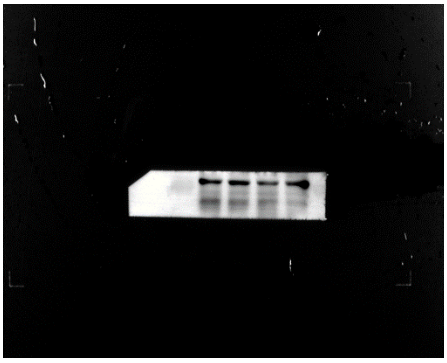

90KD

β-ACTIN

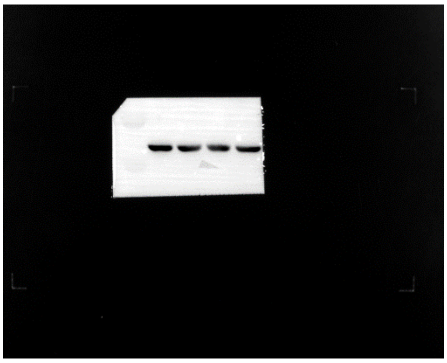

42KD

CD68

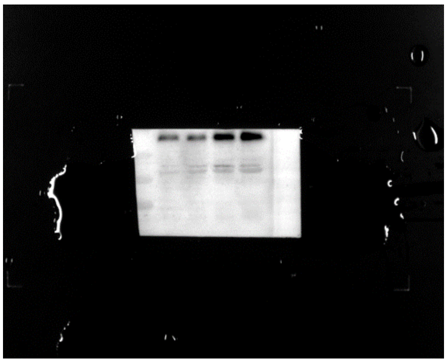

37KD

Arg1

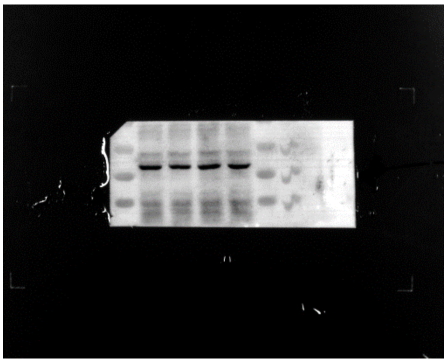

40KD

CD16
3

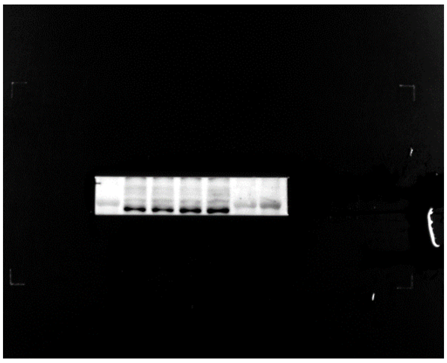

130KD

Fig S1

GAPDH

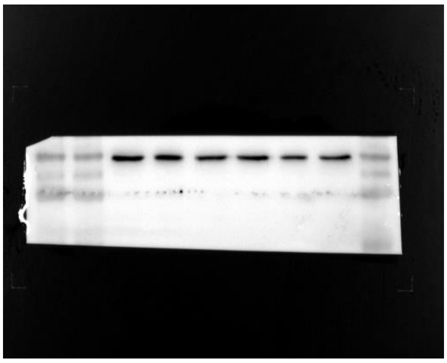

36KD

Pro-IL1β

Sec-IL1β

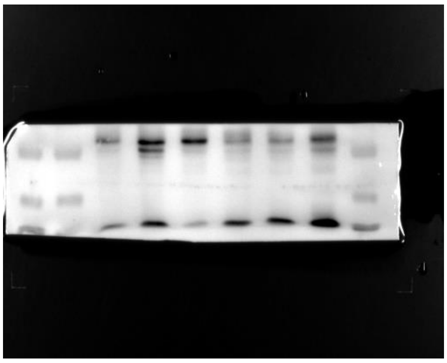

31KD

17KD

Fig

S3 b

GPX4

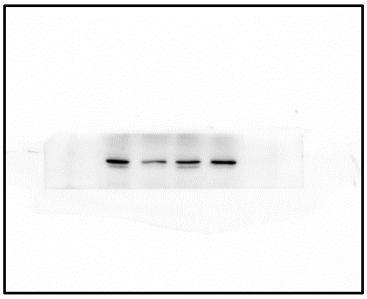

22KD

β-ACTIN

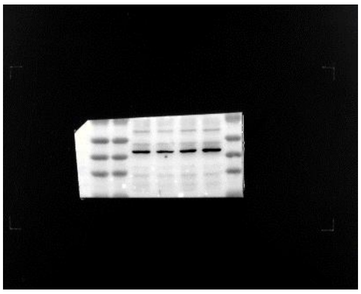

42KD

SLC7A11

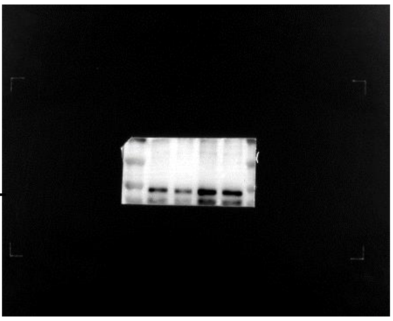

55KD

NRF2

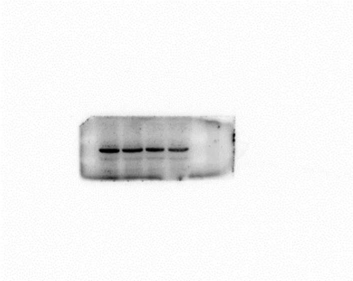

67KD

Fig.5 b

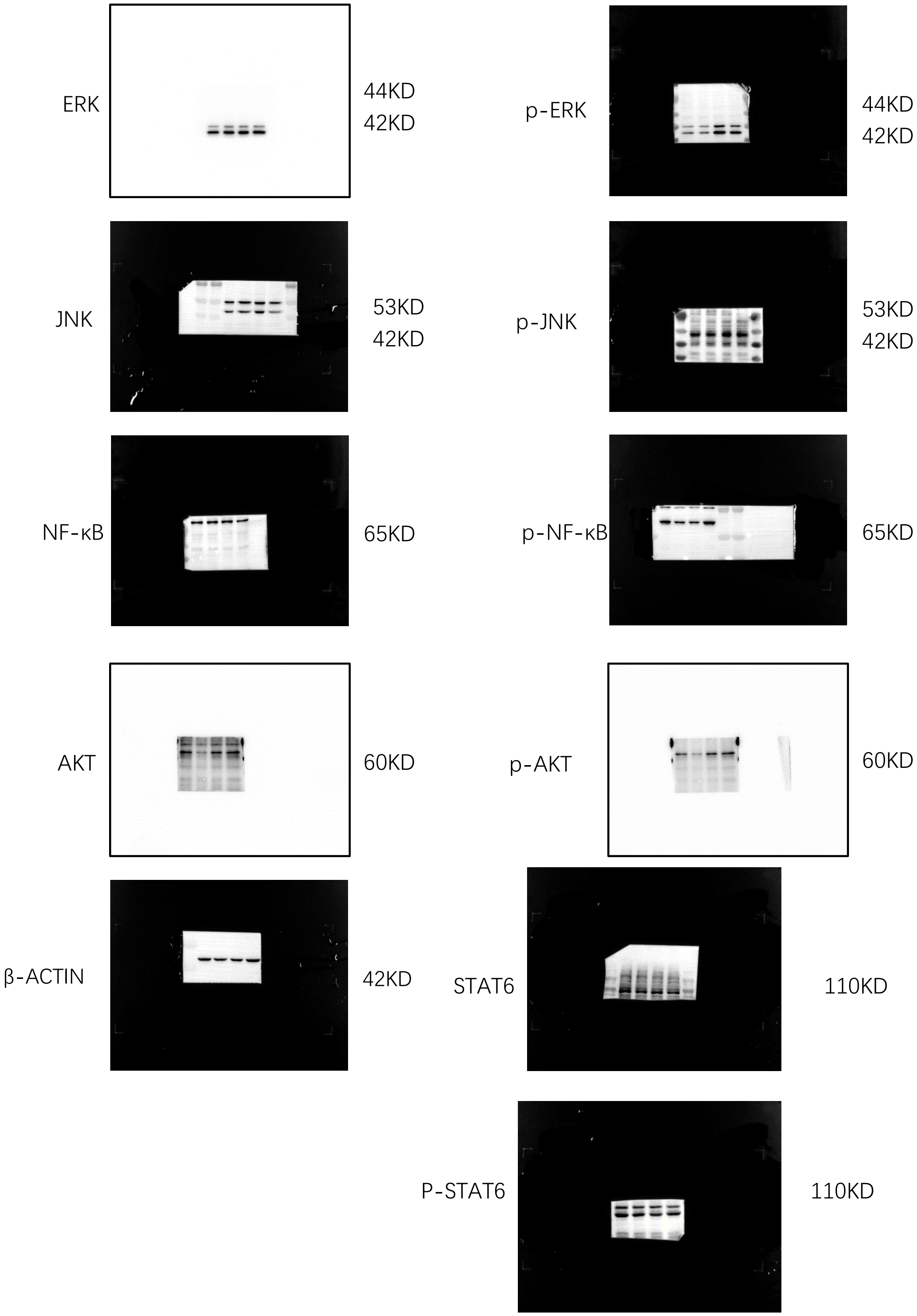
ERK
44KD
42KD
p-ERK
44KD
42KD
JNK
53KD
42KD
p-JNK
53KD
42KD
NF-κB
65KD
p-NF-κB
65KD
AKT
60KD
p-AKT
60KD
β-ACTIN
42KD
STAT6
110KD
P-STAT6
110KD

Fig.3 i

CCR6

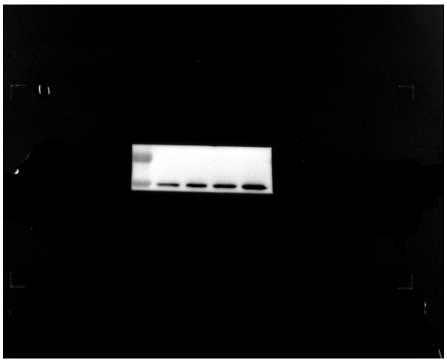

42KD

β-ACTIN

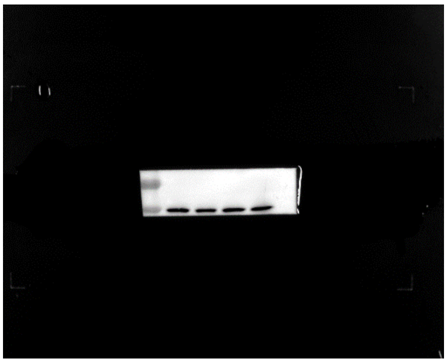

42KD

Fig.2

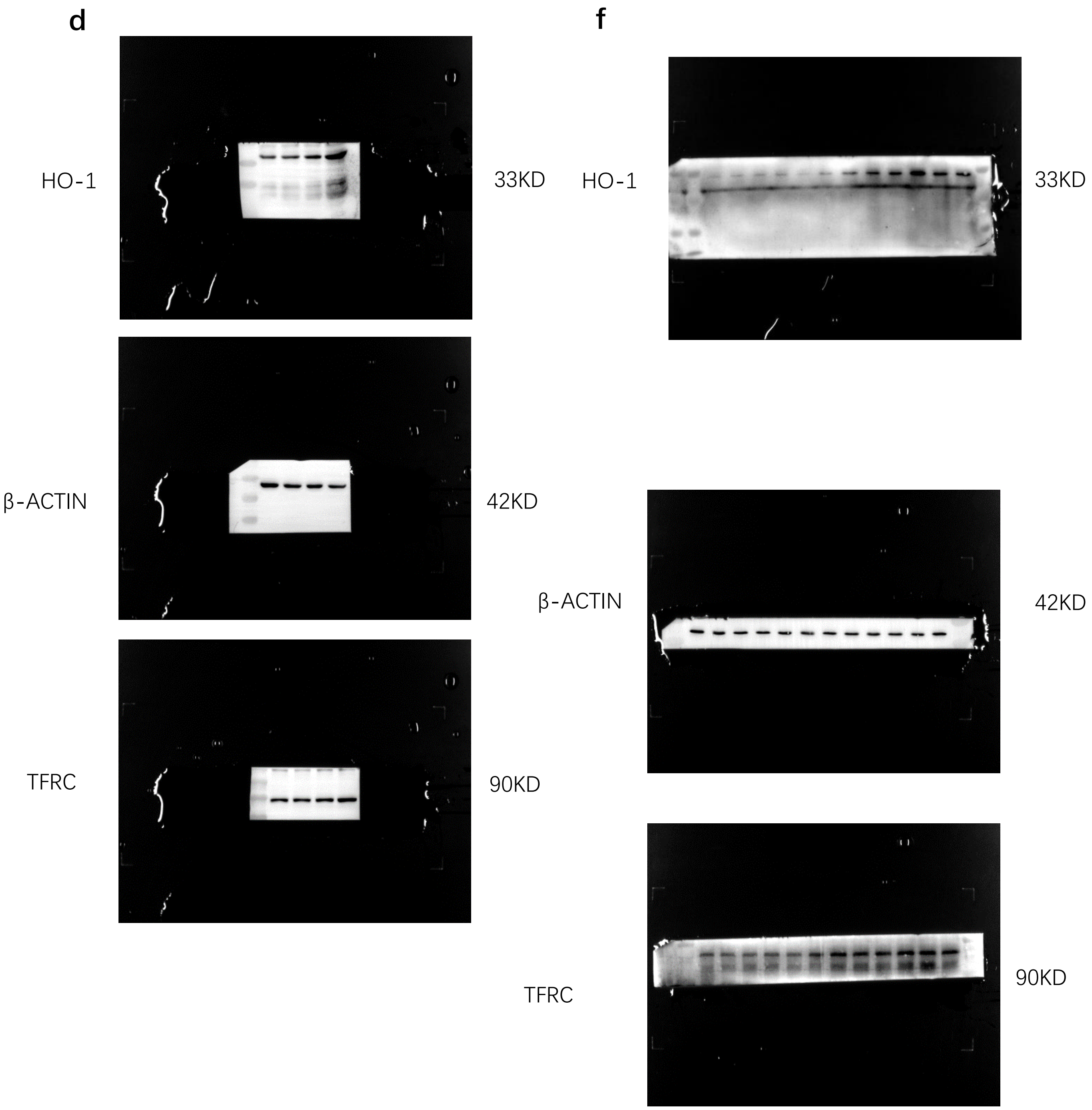
d
HO-1
33KD
β-ACTIN
42KD
TFRC
90KD
f
HO-1
33KD
β-ACTIN
42KD
TFRC
90KD

Fig S4 e

β-ACTIN

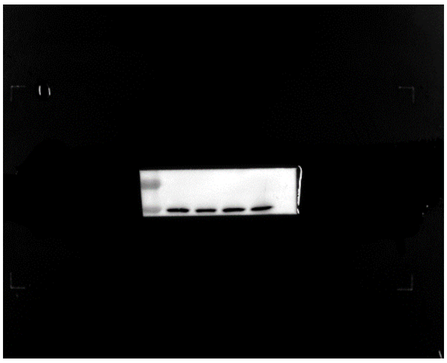

42KD

CCR5

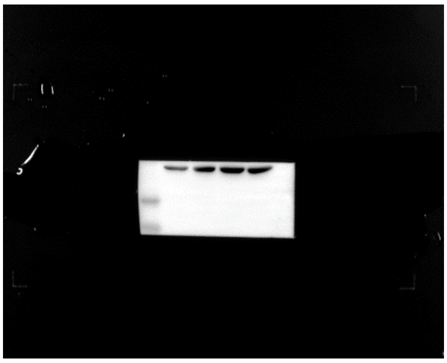

40KD
